# *In Vitro* Morphogenesis of *Arabidopsis* to Search for Novel Endophytic Fungi Modulating Plant Growth

**DOI:** 10.1371/journal.pone.0143353

**Published:** 2015-12-07

**Authors:** Francesco Dovana, Marco Mucciarelli, Maurizio Mascarello, Anna Fusconi

**Affiliations:** 1 Department of Sciences and Innovative Technology, University of Piemonte Orientale, Alessandria, Italy; 2 Department of Life Sciences and Systems Biology, University of Torino, Torino, Italy; University of Milano Bicocca, ITALY

## Abstract

Fungal endophytes have shown to affect plant growth and to confer stress tolerance to the host; however, effects of endophytes isolated from water plants have been poorly investigated. In this study, fungi isolated from stems (stem-E) and roots (root-E) of *Mentha aquatica* L. (water mint) were identified, and their morphogenetic properties analysed on *in vitro* cultured *Arabidopsis* (L.) Heynh., 14 and 21 days after inoculation (DAI). Nineteen fungi were analysed and, based on ITS analysis, 17 isolates showed to be genetically distinct. The overall effect of water mint endophytes on *Arabidopsis* fresh (FW) and dry weight (DW) was neutral and positive, respectively, and the increased DW, mainly occurring 14 DAI, was possibly related to plant defence mechanism. Only three fungi increased both FW and DW of *Arabidopsis* at 14 and 21 DAI, thus behaving as plant growth promoting (PGP) fungi. E-treatment caused a reduction of root depth and primary root length in most cases and inhibition-to-promotion of root area and lateral root length, from 14 DAI. Only *Phoma macrostoma*, among the water mint PGP fungi, increased both root area and depth, 21 DAI. Root depth and area 14 DAI were shown to influence DWs, indicating that the extension of the root system, and thus nutrient uptake, was an important determinant of plant dry biomass. Reduction of *Arabidopsis* root depth occurred to a great extent when plants where treated with stem-E while root area decreased or increased under the effects of stem-E and root-E, respectively, pointing to an influence of the endophyte origin on root extension. *M*. *aquatica* and many other perennial hydrophytes have growing worldwide application in water pollution remediation. The present study provided a model for directed screening of endophytes able to modulate plant growth in the perspective of future field applications of these fungi.

## Introduction

Plants are sessile organisms characterized by developmental plasticity, which allows them to adapt to environmental conditions. In recent years, it has become clear that plants do not live alone, but are a component of the “holobiont”, “the host organism and all its symbiotic microbiota” [1] with associated microorganisms having a remarkable role in plant adaptation and survival [1]. A large group of plant-associated microorganisms is represented by endophytic fungi (Petrini 1986, see e.g. [2]) which, in natural ecosystems are hosted by most or, perhaps, all plants. Fungal endophytes may be mycorrhizal or non-mycorrhizal, the latter are primarily formed up by Ascomycota and include the clavicipitaceous endophytes of grasses and the more heterogeneous group of the nonclavicipitaceous (NC) endophytes [2]. According to Brundrett [3], mycorrhizal associations differ from the non-mycorrhizal ones because of the construction of a specialized interface to transfer nutrients between hosts. Moreover, whilst the development of mycorrhizal fungi is restricted to roots, non-mycorrhizal endophytes may grow into the roots (e.g. the dark septate endophytes (DSE) [4]) or the stem-leaf system, or both [2].

Diversity and abundance of non-mycorrhizal endophytes is very high, even in the same population, plant and organ [2], and become enormous when considering the number of fungal strains of each species. However, despite their widespread occurrence, ecological role and the benefits of endophytic associations are still poorly understood, the responses of plants spanning from negative to positive. These latter, which mainly consist in increased stress tolerance towards biotic and abiotic stresses and plant growth, have generally been ascribed to modulation of nutrient uptake, plant phytohormones and antioxidant responses [5–7]. Moreover, some NC endophytes, growing in a stressful environment, have shown to confer habitat-adapted benefits to the host growing under the same, but not other, type of stress [2].

Endophytes have been mostly studied in terrestrial plants and their occurrence in water environments, as well as their effects on the aquatic hosts, is comparatively less known [8].

In the present study, a culture-dependent isolation method was applied to analyse *in planta* the effects of fungal endophytes inhabiting shoots and submerged roots of water mint (*Mentha aquatica*). This plant is a facultative hydrophyte, which has been used in monitoring water ecosystems quality [9]. It is a good candidate for water phytodepuration in constructed wetlands because it presents large root surface supporting the growth of beneficial microbes and secretes substances into the rhizosphere that have shown to inhibit coliform bacteria [10]. Besides, water mint tolerates prolonged dry conditions typical of seasonally flooded wetlands and intermittent streams [11].

Because little is known about growth and development of water mint *in vitro*, we assessed the effects of its endophytes on *in vitro* cultured *Arabidopsis thaliana* to address the following questions: (1) are the overall effects of water mint endophytes on plant growth positive, neutral or negative? (2) is plant biomass influenced by the fungal-related root phenotype? and (3) does *Arabidopsis* growth response differ between endophytes isolated from roots and shoots?

The use of a non-host plant for this study was justified by the intrinsic characteristics of *Arabidopsis* and by its susceptibility to be colonized by a large variety of non-mycorrhizal symbiotic microorganisms [12–15] thus becoming a model plant to investigate endophytic interactions [4, 16–19]. Moreover, almost for the DSE, the results obtained on model and native plants have shown to be similar [4].

## Materials and Methods

### Fungal endophytes isolation

Fungal endophytes were isolated from stems (stem-E) and roots (root-E) of 20 individuals of *M*. *aquatica* growing in a water stream siding Demonte river, in the Valle Stura di Demonte, Cuneo, Italy (44°18.350′N, 7°22.296′E; 680 m a.s.l.). No specific permissions were required to take samples of *M*. *aquatica* at this location; the collection of live specimens was limited to five individuals per person a day as regulated by Regional legislation (Piedmont, Italy). The field studies did not involve endangered or protected species. Three pieces (4–5 cm long) for each plant and type of organ were washed under running tap water for at least two h and then incubated for 1 h in a 4% PPM^™^ (v/v) water solution. Hereafter, stem explants were sterilized with 70% ethanol for 90 s, and 40% bleach plus 0.01% Tween 20 for 5 min; roots were sterilized with 95% ethanol for 30 s, 6% bleach plus 0.01% Tween 20 for 2 min and 2% chloramine T (w/v) in water plus 0.01% Tween 20 for 10 min. After five washes in distilled water, stems and roots pieces were cut in 10 mm-long segments, plated on Malt Extract Agar (MEA) medium and incubated at 23±1°C. Fungal colonies were isolated in pure cultures collecting aerial mycelia and classified according their morphology and growth rate in order to screen for different isolates. An imprint of the sterilized root or stem tissue was made on Potato Dextrose Agar (PDA) medium to check for effectiveness of sterilization. Water mint endophytes were grown and maintained on MEA medium and subcultured regularly.

### Molecular identification of endophytic fungi

Fungal mycelia were scraped from pure cultures grown on MEA medium for 2 weeks at 25°C in the dark and ground to a fine powder with liquid nitrogen using a mortar and pestle. Total DNA was extracted using the Qiagen Mini Kit following manufacturer’s instructions. The ITS region was amplified with primers ITS1F/ITS1 [20] and ITS4 [21]. PCR was performed in 25 μl reaction volume following [20]. The PCR products were purified and sequenced by Macrogen Inc. (Amsterdam, Europe). Sequences were assembled and edited in Geneious v. 8.1.2 [22] and then submitted to GenBank. Blast database searches were performed with ITS-fragments queries to reveal relationships to published sequences.

### Plant material and growth conditions

Endophytes were evaluated *in vitro* for their effects on *Arabidopsis* Col-0 ecotype. Seeds were surface sterilized with 75% ethanol for 90 s and 10% bleach plus 0.01% Tween 20 for 5 min. After five washes in distilled water, seeds were sown and grown on square agar plates (120×120×17 mm) containing 0.2×MS medium [23] with the addition of 0.5% myo-inositol (w/v), 0.02% glycine (w/v), 0.5% sucrose (w/v) (pH corrected to 5.7 with NaOH) and incubated at 7±1°C for 72 h. Thereafter, plants were placed in a plant growth chamber with a photoperiod of 18 h of light/6 h darkness, light intensity of 150 μmol m^-2^ s^-2^, and temperature of 23±1°C. Plates were placed at an angle of 70° to allow root growth along the agar surface and to prompt aerial growth of the hypocotyls.

### Plant and fungal co-cultures

Two mycelial plugs (7 mm diameter) cut with a sterile cork borer in non-sporulating fungal cultures were aseptically placed at a 7 cm distance from the root tip of 4-d-old germinated *Arabidopsis* seedlings (13 seedlings per plate). Exact plugs positioning was determined in a previous experiment to avoid as much as possible any contact between fungal mycelia and growing plants. In the case of sporulating isolates of *Penicillium*, fungal spore density of 10^6^ were inoculated with a sterile pipette inside two holes cored in the agar medium (50 μl each) in the same position as the mycelial plugs. Plates were doubled sealed with Parafilm. E-treated plants consisted of eight plates for each endophyte, cultured for and analysed at two different sampling periods: 14 and 21 DAI. Control plates were inoculated with plugs of MEA medium or 100 μl sterile water. Endophyte-treated plants (E-treatments) and controls were cultured at the same conditions used for germination and analysed 14 and 21 DAI.

### Plant fresh and dry weights

Four groups of three plants were measured for each treatment. Immediately after harvest, plants were blotted dry on a paper towel to remove excess of agar and water, and fresh weight (FW) measured on an analytical scale. Plant dry weights (DW) were obtained after drying plant material in a ventilated oven at 60°C to a constant weight. DWs were measured after allowing plant material to cool down to room temperature inside a desiccator. Percentage of dry-to-fresh mass were also calculated as the % ration between FWs and DWs.

### Root system morphology

Images of the whole plants were acquired with an Epson Perfection V300 scanner (Epson America, USA) at 600 dpi, using Adobe Photoshop software (Adobe Systems, USA) and saved in TIFF format. At each sampling time, to evaluate the capacity of the root system to explore the growth medium, the root area and the root depth were determined. The root area was determined as the total root surface included in a rectangular frame having a 20 mm width; five frames for each treatment were analysed. Within each frame, root depth was measured as the length of projection on a Y axes of the distance between the root collar and the more distal root apex (S1 Fig). Images were processed with ImageJ 1.48v.

Root system architecture (RSA) was determined 14 DAI on E-treatments showing significant alterations in FW and/or DW in relation to controls and at both sampling times. The total number and length of 1^st^ order lateral roots, and the length of the primary root were measured in 6 plants per treatment. Branching of the primary root was calculated as the ratio between the number of emerged laterals and primary root length (mm).

### Statistical analysis

Data variability and comparison with controls were represented by using boxplots drawn in R (version 3.1.2). Variability of aggregated values of plant fresh and dry weights, % dry weights, root areas and root depths were also presented for stem endophytes (stem-E), root endophytes (root-E) and all fungi (E). The significance of differences between the control and E-treatments was statistically evaluated by ANOVA with Dunnett’s test for multiple comparison of means implemented in R package *multcomp*. Differences were considered significant at a probability level of p<0.05. To equalize variances, biomasses and RSA parameters were log_10_ transformed. Percentage data of dry weights were transformed to arcsin square root percentage before analysis.

To look for correlations between root morphometric parameters and plant biomasses, linear regression analysis (adjusted R^2^) on mean values at 14 and 21 DAI were performed.

## Results

### Molecular identification of water mint endophytes

Nineteen isolates were chosen for their morphological and growth characteristics. ITS sequence data analysis led to the genetic differentiation of 17 isolates, among these, nine fungi showed a 100% identity with sequences deposited in GenBank (Table 1) and corresponded to the following species: *Aureobasidium pullulans* (de Bary & Löwenthal) G. Arnaud (SE), *Cadophora luteo-olivacea* (J.F.H. Beyma) T.C. Harr. & McNew (SA, SL), *Cladosporium halotolerans* Zalar, de Hoog & Gunde-Cim. (ST2), *Colletotrichum destructivum* O'Gara (SL23), *Nemania serpens* (Pers.) Gray (RT6c), *Penicillium resedanum* McLennan & Ducker (RL3), *Penicillium solitum* Westling (RT5a), *Sarocladium strictum* (W. Gams) Summerb. (SS).

**Table 1:** Closest match of fungal isolate ITS sequence inferred from Blastn search in GenBank.

Fungal acronyms	Organ source of isolation	BLASTn closest match (Accession No.)	ITS length (query/reference) (Similarity, %)	GenBank Accessions of fungi from this study
SA	stem	*Cadophora luteo-olivacea* (GQ214536)	626/626 (100%)	KU141395
SB	stem	*Phoma macrostoma* (GU237740)	484/485 (99%)	KU141382
SE	stem	*Aureobasidium pullulans* (FN868454)	600/600 (100%)	KU141396
SL	stem	*Cadophora luteo-olivacea* (GQ214536)	607/607 (100%)	KU141394
SL23	stem	*Colletotrichum destructivum* (JQ005764)	518/518 (100%)	KU141392
SO	stem	Pleosporaceae sp. (KF636768)	554/555 (99%)	KU141381
ST2	stem	*Cladosporium halotolerans* (LN834365)	549/549 (100%)	KU141393
ST3	stem	Pleosporales sp. (FN548157)	598/600 (99%)	KU141380
SS	stem	*Sarocladium strictum* (KC311519)	553/553 (100%)	KU141379
RL3	root	*Penicillium resedanum* (JN689345)	580/580 (100%)	KU141384
RL6	root	Fungal sp. (HM123626)	572/648 (88%)	KU141390
RT5a	root	*Penicillium solitum* (JN642222)	547/547 (100%)	KU141383
RT5b	root	*Ophiosphaerella narmari* (KP690979)	452/495 (91%)	KU141388
RT6c	root	*Nemania serpens* (EF155504)	600/600 (100%)	KU141386
RT9	root	*Nemania serpens* (EF155504)	602/603 (99%)	KU141385
RT9b	root	*Chaetomium funicola* (EU552109)	545/555 (98%)	KU141378
RT10	root	Mucoromycotina sp. (HQ406814)	372/408 (91%)	KU141387
RT13	root	*Cercophora coprophila* (AY999136)	489/528 (93%)	KU141391
RT14	root	Mucoromycotina sp. (HQ406814)	372/408 (91%)	KU141389

Two fungi, *Cadophora luteo-olivacea* (SA and SL) and *Nemania serpens* (RT6c and RT9) included two isolates each (Table 1). The ITS sequence of isolate RT9 differed only by one nucleotide from *N*. *serpens* RT6c isolate and was considered the same species. In the case of *P*. *solitum* and related taxa, the ITS region is highly conserved [24] thus the precise attribution at the species level of the isolate RT5a is still under study.

### Plant fresh and dry weights

**Fig 1:**
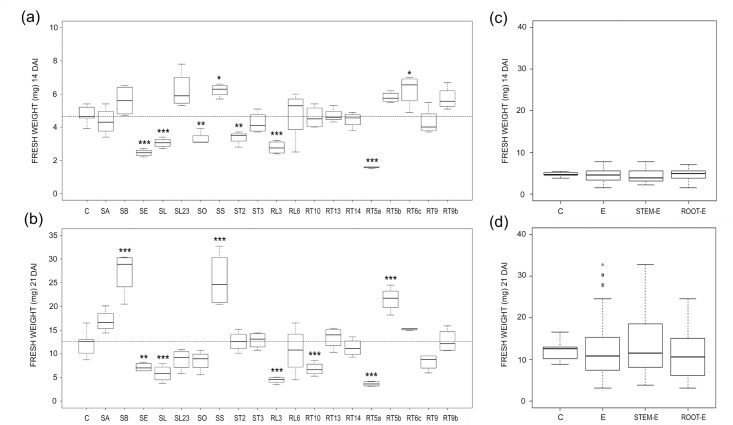
Endophyte effects on fresh weights. (a, b) Boxplots illustrating variability of fresh weight in E-treated and control (C) *Arabidopsis* plants 14 (a) and 21 (b) DAI. The reference hatched line represents the median of controls. Differences were considered significant at a probability level of *: p<0.05; **: <0.01; and ***: <0.001. (c, d) Pooled data for controls (C) and plants treated with all (E), stem (stem-E) and root (root-E) endophytes and 14 (a) and 21 (b) DAI.

The effects of the different endophytes on *Arabidopsis* FW varied considerably, and ranged from significant plant promotion to inhibition, at both 14 and 21 DAI (Fig 1a and 1b).

Co-culture of *Arabidopsis* with the fungal isolate SS and RT6c increased the FW of about 30% in relation to the controls, while SE, SL, SO, ST2, RL3 and RT5a decreased significantly it (Fig 1a). The highest inhibition, of about 67%, was induced by the fungal isolate RT5a, while the reduction of plant biomasses due to the other fungi ranged between 29 and 49%.

Only a few fungal endophytes which significantly affected FW 14 DAI, affected it also 21 DAI; they were: SS, which significantly increased FW, and SE, SL, RL3 and RT5a, which negatively affected it (Fig 1b). Some fungal isolates significantly increased (SB and RT5b) or decreased (RT10) FW 21 DAI only (Fig 1b). A significant correlation was found between FWs of the 1^st^ and 2^nd^ sampling time (R^2^ = 0.396; P = 0.002) and the analysis of pooled data on FW did not indicate any significant difference between controls and E-treated plants, despite a slight decrease was found in relation to controls (Fig 1c and 1d).

**Fig 2:**
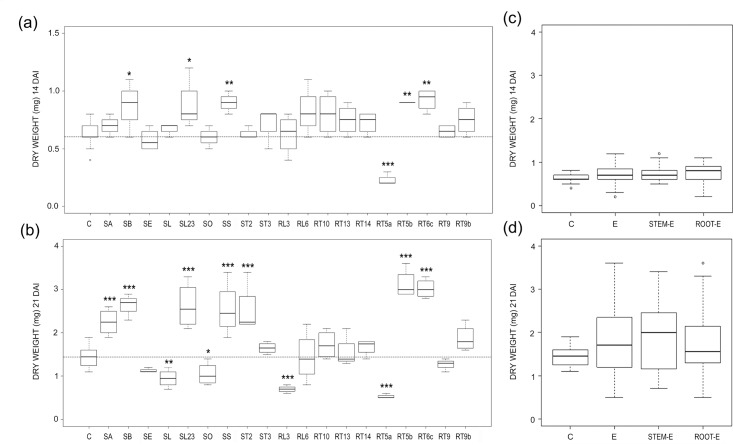
Endophyte effects on dry weights. (a, b) Boxplots illustrating variability of dry weight in E-treated and control (C) *Arabidopsis* plants 14 (a) and 21 (b) DAI. The reference hatched line represents the median of controls. Differences were considered significant at a probability level of *: p<0.05; **: <0.01; and ***: <0.001. (c, d) Pooled data for controls (C) and plants treated with all (E), stem (stem-E) and root (root-E) endophytes and 14 (a) and 21 (b) DAI.

Water mint endophytes affected *Arabidopsis* DW in a different manner. In fact, most fungal isolates increased significantly or had a neutral effect on DW, 14 and 21 DAI (Fig 2a and 2b). This led to a general increase in DW as confirmed in the analysis of pooled data (Fig 2c and 2d). All isolates that significantly increased DW 14 DAI (SB, SL23, SS, RT5b and RT6c) showed the same effect 21 DAI, others (SA and SS) increased significantly DW 21 DAI only (Fig 2a and 2b). Except for the isolate RT5a none of the fungal endophytes significantly decreased the DW of *Arabidopsis* 14 DAI, while three isolates in addition to RT5a, namely SL, SO and RL3, reduced significantly plant DW 21 DAI. Data obtained 21 DAI were strictly correlated to those at 14 DAI (R^2^ = 0.578; P = 0.000).

Overall, both FW and DW were significantly increased by three fungi (SB, SS and RT5b) at 14 and 21 DAI, and lowered by other three (SL, RL3 and RT5a), 21 DAI (Figs 1 and 2). Among the latter, RL3 and RT5a early sporulated and the mycelium extensively grew on the roots making impossible root measurements, thus they were excluded from morphometric analysis 21 DAI.

**Fig 3:**
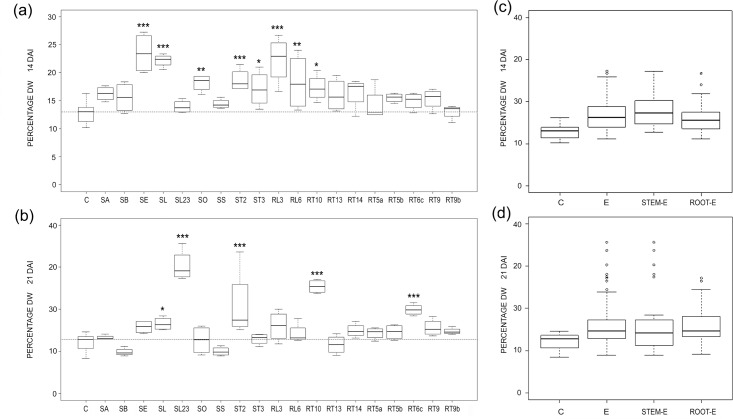
Endophyte effects on percentage dry weights. (a, b) Boxplots illustrating variability of percentage dry weight in E-treated and control (C) *Arabidopsis* plants 14 (a) and 21 (b) DAI. The reference hatched line represents the median of controls. Differences were considered significant at a probability level of *: P<0.05; **: <0.01; and ***: <0.001. (c, d) Pooled data for controls (C) and plants treated with all (E), stem (stem-E) and root (root-E) endophytes and 14 (a) and 21 (b) DAI.

The % dry-to-fresh biomass of *Arabidopsis* increased in relation to controls, and this was striking 14 DAI, when it occurred in all E-treatments, significantly in almost half of them (Fig 3a); most effects were instead neutral 21 DAI and by this time only 5 fungal isolates caused a significant increase of the % dry-to-fresh mass of *Arabidopsis* (Fig 3b). The analysis of pooled data showed a % dry-to-fresh mass increase in E-treated plants in relation to controls by 14 DAI (Fig 3c and 3d).

The two isolates of *C*. *luteo-olivacea* (SA and SL) showed a different effect on *Arabidopsis* weights. The fungal isolate SA had little effects on these parameters, showing a significant DW increase 21 DAI only, and neutral effect on both fresh and % dry-to-fresh biomass; on the contrary, SL decreased significantly FW 14 and 21 DAI, and DW 21 day, leading to a significant increase of the % dry-to-fresh biomass at both sampling times.

### Extension of the root system: root area and root depth

**Fig 4:**
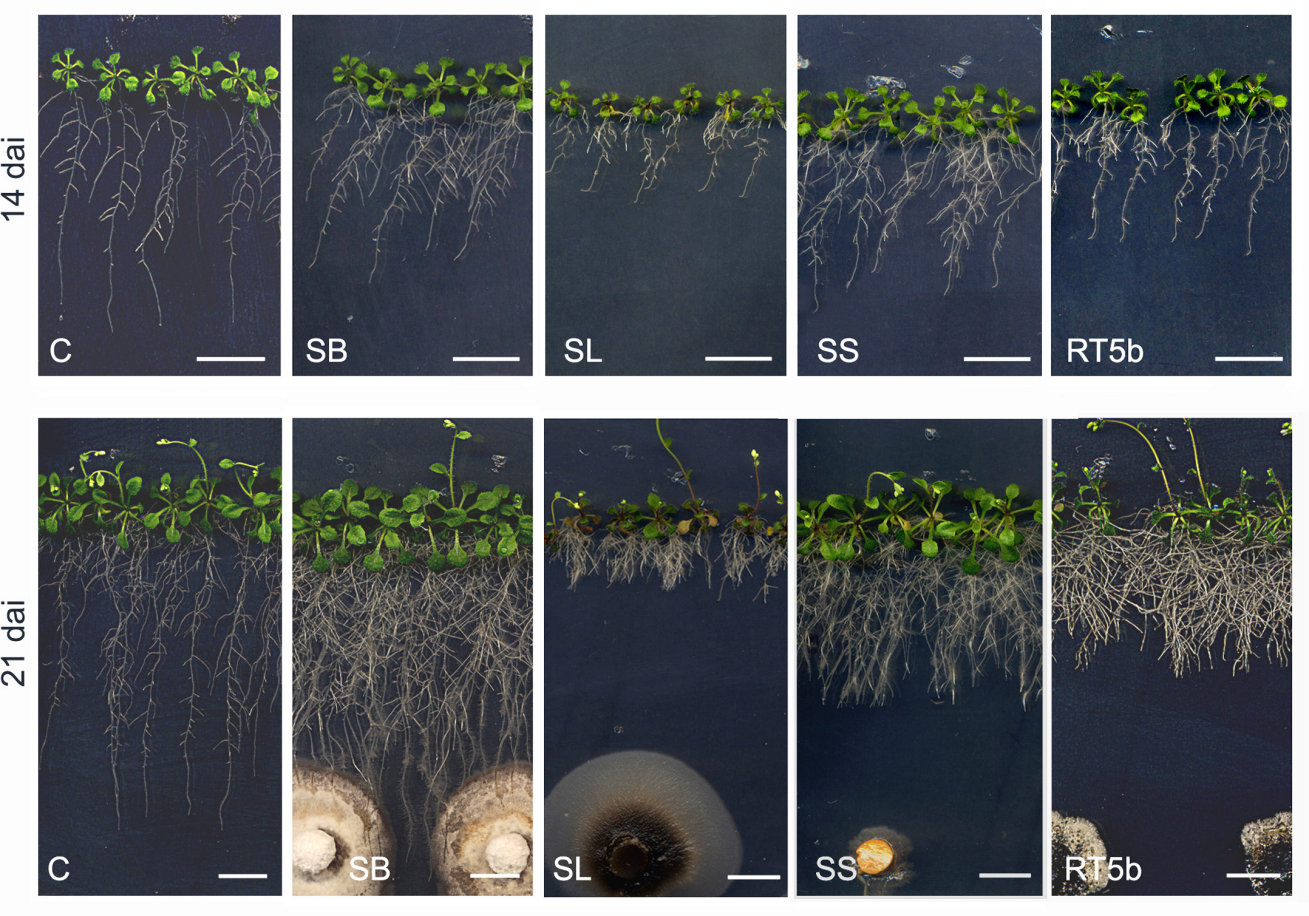
Endophyte-related root phenotypes in *Arabidopsis*. Plants and fungus co-cultures 14 DAI (top row, a-f) and 21 DAI (bottom row, a’-f’).

**Fig 5:**
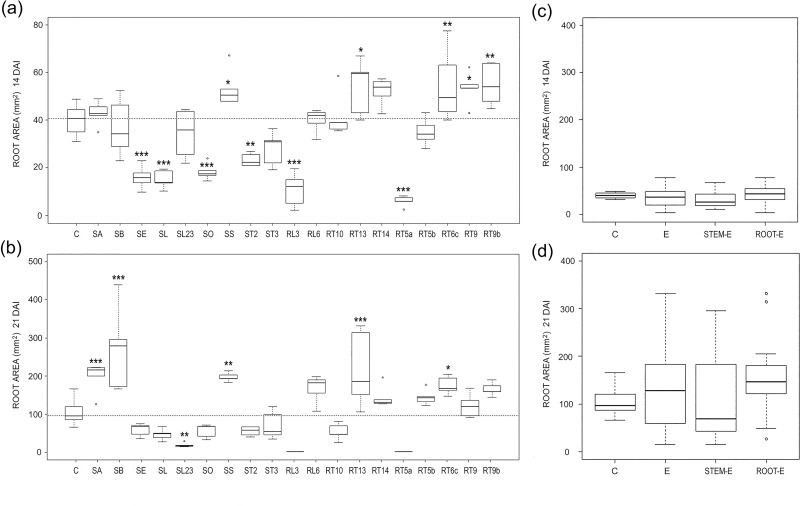
Endophyte effects on root areas. (a, b) Boxplots illustrating root area variability in E-treated and control (C) *Arabidopsis* plants 14 (a) and 21 (b) DAI. The reference hatched line represents the median of controls. Differences were considered significant at a probability level of *: p<0.05; **: <0.01; and ***: <0.001. (c, d) Pooled data for controls (C) and plants treated with all (E), stem (stem-E) and root (root-E) endophytes and 14 (a) and 21 (b) DAI.

*Arabidopsis* root system extension changed considerably and significantly 14 and 21 DAI with water mint endophytes (Fig 4). Six isolates decreased root area significantly, while five significantly increased it, 14 DAI (Fig 5a). At the end of the experiment, although the number of stimulating or repressing fungi was about the same, only SL23 decreased significantly root area (Fig 5b). Five fungi significantly increased root area 21 DAI, including SB (Fig 4b and 4b’) and SS (Fig 4d and 4d’), which significantly increased FW and DW at both sampling times. An increase of root area in relation to controls, i.e. a reduction of the negative effects or an increase of the positive ones, was found between the 1^st^ and 2^nd^ samplings in most E-treatments (Fig 5a and 5b). This trend was confirmed by the analysis of pooled data. Stem-E reduced *Arabidopsis* root area, while root-E tended to increase it (Fig 5c and 5d).

**Fig 6:**
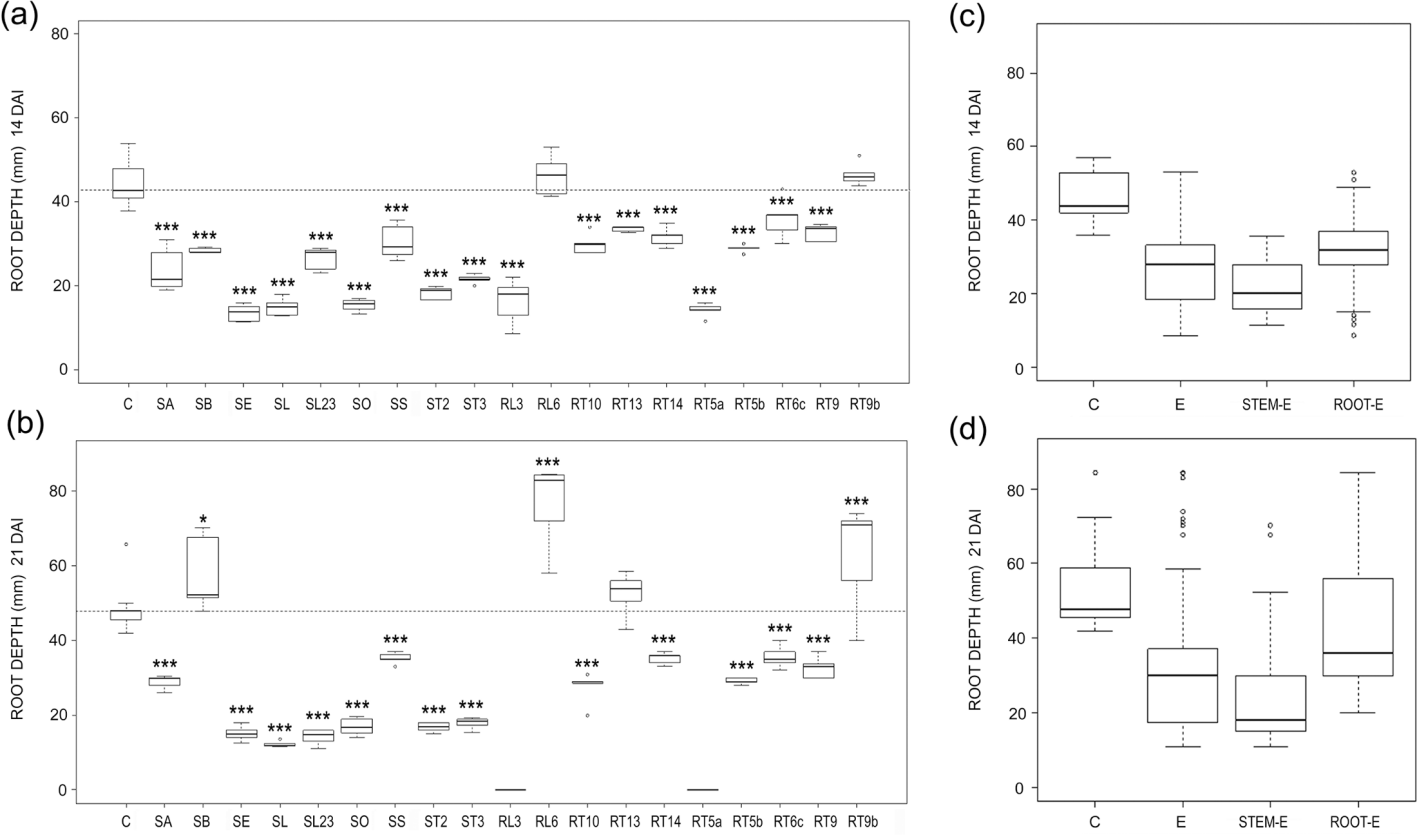
Endophyte effects on root depth. (a, b) Boxplots illustrating variability of root depth in E-treated and control (C) *Arabidopsis* plants 14 (a) and 21 (b) DAI. The reference hatched line represents the median of controls. Differences were considered significant at a probability level of *: p<0.05; **: <0.01; and ***: <0.001. (c, d) Pooled data for controls (C) and plants treated with all (E), stem (stem-E) and root (root-E) endophytes and 14 (a) and 21 (b) DAI.

On the contrary, a consistent, significant decrease of root depth with respect to controls occurred, at both samplings and in almost all E-treated plants (Figs 4c–4e, 4c’–4e’ and 6a and 6b). *Arabidopsis* root apparatus depth was significantly decreased 14 DAI by almost all water mint fungi (Fig 6a). Root depth increased only slightly between 14 and 21 DAI with most fungi and 21 DAI all isolates except four continued to significantly reduce the parameter (Fig 6b). Exceptions were represented by fungal isolates RL6 and RT9b, whose positive effect become significant 21 DAI only, and by SB (Fig 4b’) which increased significantly the parameter (Fig 6b). Accordingly, the analysis of pooled data showed a decrease of root depth in E-treated plants at both samplings, which was more pronounced when stem-E fungi were considered (Fig 6c and 6d).

**Fig 7:**
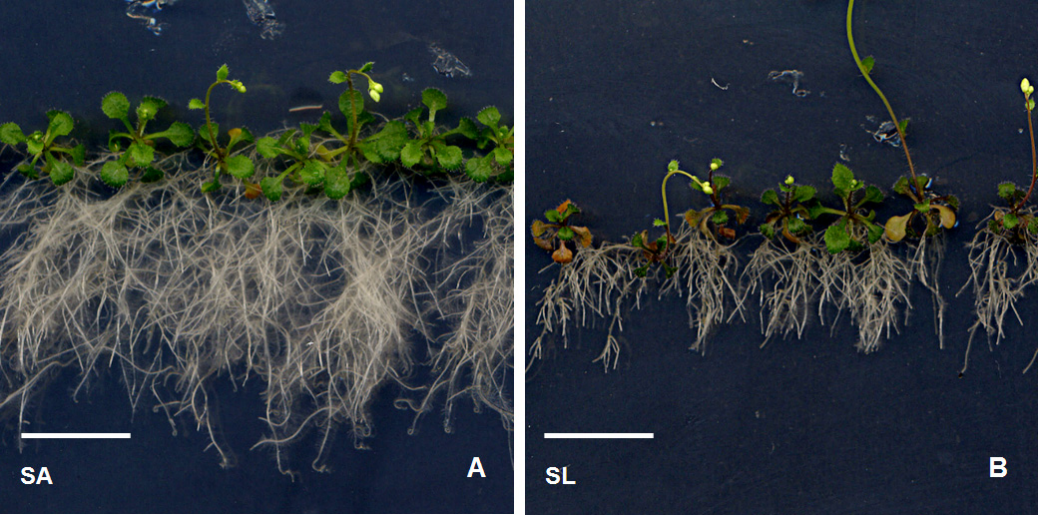
*Cadophora luteo-olivacea* related root phenotypes in *Arabidopsis*. Plants and fungus co-cultures 21 DAI with SA (a) and SL (b).

Both isolates of *C*. *luteo-olivacea* strongly reduced root depth (Fig 6a and 6b), however, SA increased root area, significantly 21 DAI, while SL showed a repressive effect on this parameter, significant 14 DAI (Figs 5a and 5b and 7a and 7b).

### Root system architecture (RSA)

The morphometric parameters related to RSA of *Arabidopsis* were analysed 14 DAI on ten E-treatments that were characterized by coherent and significant alterations in FW and/or DW in relation to controls at both samplings. The fungal isolate SA of *C*. *luteo-olivacea* was added to be compared with SL, of the same species. The selected isolates were: SA, SB, SE, SL, SL23, SS, RL3, RT5a, RT5b and RT6c.

**Fig 8:**
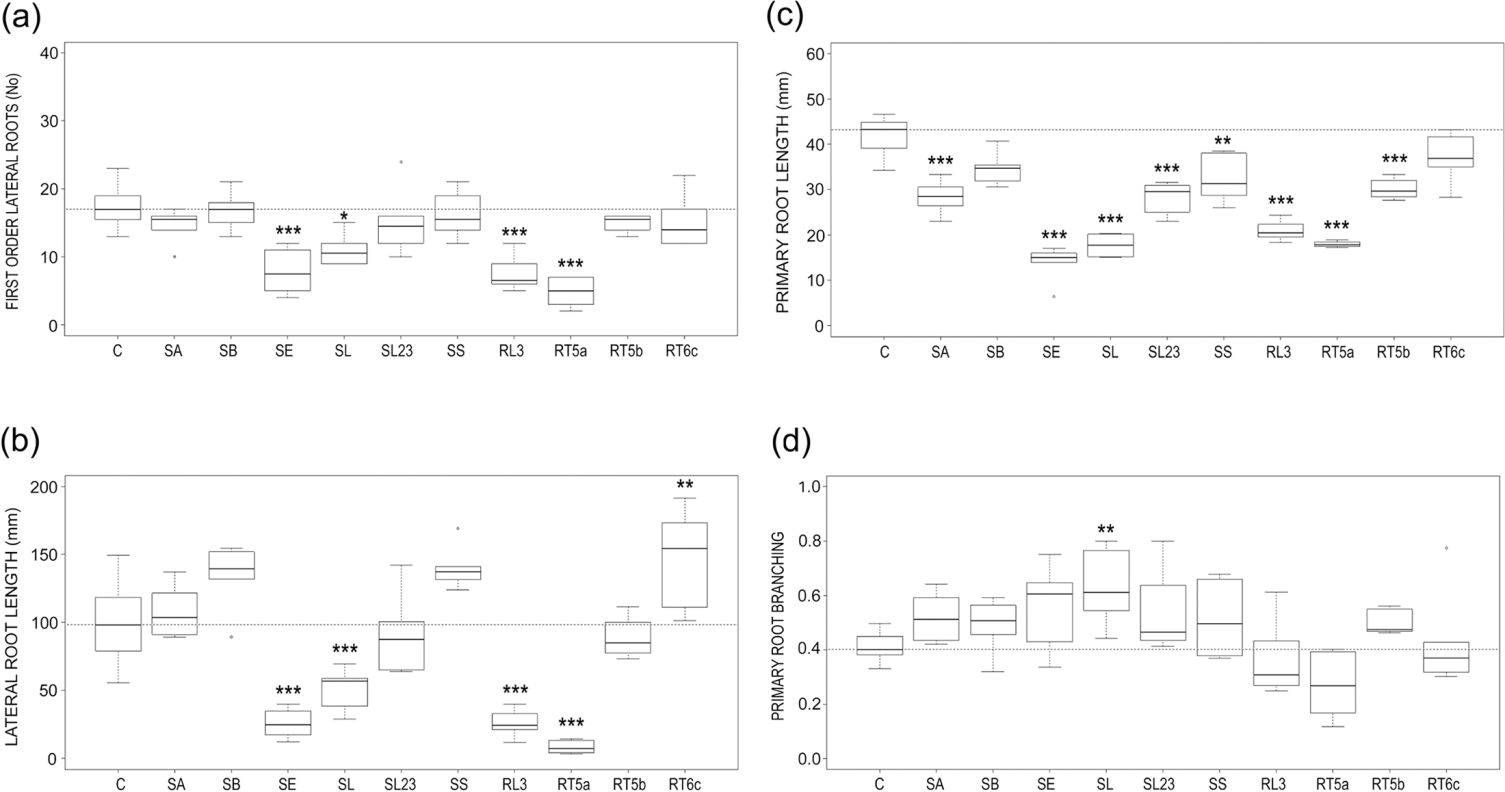
Endophyte effects on root system architecture. Boxplots illustrating root system architecture variability in E-treated and control (C) *Arabidopsis* plants, 14 DAI. (a) number of first order lateral root; (b) total length of lateral root; (c) primary root length and (d) primary root branching. The reference hatched line represents the median of controls. Differences were considered significant at a probability level of *: p<0.05; **: <0.01; and ***: <0.001.

None of the selected endophytes increased the number of first order lateral roots of *Arabidopsis* with respect to the control plants, and decreases were significant for four of them: SE, SL, RL3 and RT5a (Fig 8a). The same four fungi reduced significantly, between 51 and 81%, the total lateral root length of *Arabidopsis*, while RT6c significantly increased it (Fig 8b). A substantial reduction of the length of the primary root occurred in all E-treatments of *Arabidopsis*; this decrease was almost always significant, and ranged between 23 and 67%; only SB and RT6c caused a non-significant reduction of this parameter (Fig 8c). Variations in primary and total lateral root lengths in relation to controls were tightly related to those found for root depth and root area, respectively (adjusted R^2^ = 0.93; p<0.001; adjusted R^2^ = 0.92; p<0.001).

The general reduction in both the primary root length and in the number of lateral roots led to a not-significant effect on the primary root branching of *Arabidopsis* in most cases. In fact, lateral root density of the primary root was modified significantly only by the SL fungal isolate of *C*. *luteo-olivacea*, which increased it due to the strong shortening of the primary root (Figs 4c and 8d).

Interestingly, fungal isolates SL and SA of *C*. *luteo-olivacea* modified the number of lateral roots and primary root length of *Arabidopsis* with the same trend, by reducing them, and both had a positive effect on root branching. However, the effects exerted by SL were comparatively higher and only SL reduced significantly the lateral root length (Figs 7 and 8a–8d).

## Discussion

In this study, for the first time, the effects of number of endophytes isolated from healthy stems and submerged roots of *M*. *aquatica* L. (water mint) were analyzed in order to compare and comprehensively evaluate their effects on growth and root architecture in *Arabidopsis*. Although the use of microbial inoculants naturally associated with the host plant is advisable [25], in nature *Arabidopsis* may be colonized by a very large variety of microorganisms, and it has become a recognized model to analyse non-mycorrhizal plant-microbe interactions [4, 12, 26].

### Fungal endophytes of water mint

A few of the water mint endophytes had previously been reported in other plant hosts and in different habitats. The water mint fungal isolate SE of *Aureobasidium pullulans*, for example, belongs to the NC-endophytes of class 2 *sensu* [2], as it is epiphytic and endophytic in healthy leaves, stems, roots and seeds of different host species [27]. Similarly, the fungal isolate SS of *Sarocladium strictum* is an endophyte in the roots of several medicinal plants [28], and in stems of *Salicornia europea* [29].

Other water mint endophytes are ecologically more variable being also described in literature as saprobes in different habitats. The fungal isolate ST2, here referred as *Cladosporium halotolerans*, is an endophyte of healthy stems and leaves of *Hypericum perforatum* but also found saprobe in salty or hypersaline environments [30].

### The effects of the fungal isolates on *Arabidopsis* growth

Fungal endophytes in culture can manifest a wide range of lifestyles, including weak pathogens and dormant saprobes. For such reason, we synchronized co-cultures of fungi and *Arabidopsis* in order to limit the physical contact between the two partners (Fig 4). Only in the case of the root promoter isolate SB (Fig 4b and 4b’) and of the sporulating fungi RT5a and RL3, contact between the fungal colony and *Arabidopsis* roots occurred by the end of experiment.

As expected, in terms of plant biomass, the effects of water mint endophytes ranged from inhibition to promotion of *Arabidopsis* growth (Figs 1a and 1b and 2a and 2b). In some cases the effects observed were consistent with the literature as it happened for *S*. *strictum* (SS) which showed a PGP effect on *Arabidopsis* (Fig 4d and 4d’) and other hosts [31–32]. On the contrary, the water mint isolate SB, genetically close to the pathogen *Phoma macrostoma* with bioherbicidal activity [33], in our study turned to be a PGP fungus (Fig 4b’). The opposite occurred with the water mint isolates SE, RT5a and RL3, which affected negatively *Arabidopsis* plant biomass, although *A*. *pullulans* was described as a biological control agent capable of beneficial effects on hosts [34, 35], and *P*. *resedanum* and *P*. *solitum* strains showed to be beneficial or neutral when associated to *Capsicum annuum* [36] and *Solanum lycopersicum* [37], respectively.

Differences between our results and those of the literature can rely on different experimental procedures. However, fungal isolates belonging to the same species may behave differently. Variability of plant host effects among different isolates of the same species has been documented in the literature for *P*. *solitum* [37, 38] and *C*. *luteo-olivacea* [39, 40]. Accordingly, in our work the two isolates of *C*. *luteo-olivacea* exerted a strong and opposite influence on *Arabidopsis* growth. In fact, SA significantly increased FW, DW and root area while SL decreased the same characters, 21 DAI (Fig 7), indicating that different isolates of the same species may behave differently even when isolated from the same plant organ.

As a whole, water mint endophytes exerted a neutral/beneficial effect on plant growth (see FW and DW pooled data, Figs 1c and 1d and 2c and 2d). This contrasts with results from a recent meta-analysis conducted on DSE, where the response of total biomass to fungal inoculation was about 18% lower than non-inoculated controls [6], despite the observed PGP effect of a number of DSE [41].

All water mint endophytes induced an increase of % dry-to-fresh biomass 14 DAI at least (Fig 3a), mainly due to a general increase in DW (Fig 2a–2d). Variation of plant biomass following fungus inoculation is frequently expressed as variations in fresh or dry weights [12, 14, 42, 43] and % dry-to-fresh biomass has been rarely reported. However, PGP endophytes such as *P*. *indica* [44] and PGP-HSF [45, 46], increased more the fresh than the dry biomasses of *Thymus vulgaris* and *Mentha piperita*, respectively, while in other cases the influence of endophytes on the % in DW and FW were rather similar [44, 47]. Thus, the increase in the DW unrelated to FW observed in our work (SL23, ST2, RT6c and RT10; compare Figs 1b and 3b) is difficult to explain. We suspected that a number of water mint isolates behaved as mild pathogens when co-cultured with *Arabidopsis*, as generally occurs during non-systemic endophyte infection [48, 49 and references therein]. In response, plants may have reduced cell elongation, thus producing more cells per volume unit, and/or increased cell wall thickness [50]). Auxin, which is known to be extremely important not only in plant development [51, 52] but also in plant-pathogen interactions and plant defence mechanisms (reviewed by [50]), could tentatively be involved in this response.

Water mint endophytes affected significantly *Arabidopsis* root shape and extension and a significant decrease of root depth with respect to control plants occurred by 14 DAI, in almost all treatments (Fig 6a and 6b). This accorded with the substantial and consistent reduction of the primary root length induced by the ten selected fungi 14 DAI (Fig 8c). Because this occurred along with a general reduction of the number of lateral roots (Fig 8a), root branching did not change significantly with most endophytes (Fig 8d).

A comparison of the effects of the different fungi on *Arabidopsis* root development 14 DAI showed that the reduction in root depth and total root area with respect to control plants was related to plant growth on a DW basis (adjusted R^2^ = 0.21; P = 0.024 and adjusted R^2^ = 0.37; P = 0.003, respectively). On the contrary, significant relations were not found among the same parameters 21 DAI. This indicates that the effect exerted by a number of endophytes on DW could depend on a root system more or less efficient in nutrient absorption in the early growth stages.

However, it is worth noting that alterations in RSA can influence plant growth independently of nutrient uptake by regulating plant-microorganism interactions. A reduction in root length, as above explained, may be related to more rigid cell walls and greater resistance towards pathogens [50]. Moreover, RSA plays a significant role in determining composition and quantity of exudates (reviewed by [53]). Water mint isolates, by changing the proportion of apical, elongating and mature root zone, may regulate the release of nutrients, gases, anti-microbial or signalling compounds, which are important chemical mediators between plant and rhizospheric microorganisms and, as already discussed, can also affect the phytodepurative properties of the plant [10].

Microbial endophytes can influence plant morphogenesis directly, by releasing auxins, or indirectly, through regulating auxin biosynthesis, homeostasis and signalling in host tissues [14, 52, 54, 55]. Because one of the most striking effect of E-treatment on *Arabidopsis* was primary root shortening, which is typically induced by high concentrations of auxin [14, 56], we estimated the presence of IAA derivatives in water mint endophytes growth medium. None of the water mint endophytes produced IAA derivatives in liquid culture, with the exclusion of SA (*C*. *luteo-olivacea*), whose IAA-derivatives level (0.055 μg/ml; S1 Text) was however lower than the range of concentrations reported for other fungal endophytes [57–59]. The response of *Arabidopsis* to SA was characterized by a significant increase of DW and root area, only 21 DAI, and by a strong reduction in the length of the primary root and root depth, without a significant branching increase (14 DAI). Therefore, growth alterations produced by SA accorded only in part with the production of auxin by the fungus [56] and were similar to those induced e.g. by SS, whose auxin levels were not detectable. This led us to suppose the influence of other factors on root development, although a more specific auxin content quantification, and the use of auxin synthesis and signaling *Arabidopsis* mutants are needed to draw any conclusion.

Plant tissues and organs represent different ecological niches with regard to endophyte diversity, favouring host-specific and organ-specific endophytes [8]. Fungal endophytes have been shown to produce and release different substances according to the tissue or organ of origin [60]. Our results pointed to a dependence of *Arabidopsis* root morphogenesis on the organ source of endophytes. In fact, although pooled data showed comparable effects of stem-E and root-E on FW (Fig 1c and 1d), the increase in dry biomasses of E-treatments was mainly ascribable to stem-E, 21 DAI (Fig 2d). Moreover, *Arabidopsis* root area decreased or increased under the effects of stem-E and root-E, respectively (Fig 5c and 5d) and reduction of root depth occurred to a great extent when plants where treated with stem-E (Fig 6c and 6d).

## Conclusions

We proposed a model for the occurrence of plant growth-modulating traits in water mint-associated fungi, contributing to elucidate the role on plant growth of fungi living in an aquatic environment.

Our analyses allowed us to recognize three PGP fungi, SB, SS and RT5b, which increased both FW and DW of *Arabidopsis* at 14 and 21 DAI. However, effects of these fungi on root extension were variable: two of them, SB and SS, increased root area significantly 21 DAI, while only SB enhanced root depth 21 DAI. In general, although *Arabidopsis* responses to inoculation varied significantly according to the fungal endophyte, with effects ranging from inhibition to promotion of plant growth, we were able to establish that: (1) *Arabidopsis* growth responses under the influence of water mint endophytes in terms of FW and DW were neutral and positive, respectively; the effect on DW addressing to a typical plant response toward non-systemic and potentially pathogenic infections; (2) a consistent decrease in root depth and primary root length were the main features of root extension modulation which influenced DWs 14 DAI; (3) Root extension was likely related to the source organ (shoot or root), although this aspect need further confirmation.

Many questions remain unresolved; nevertheless this model could be used for screening the ability of other endophytes to modulate *Arabidopsis* RSA, in the perspective of future field applications of these fungi.

Water mint is a facultative hydrophyte that, due to its relatively high biomass, fast growth and depurative capacity, is useful for phytodepuration in constructed wetlands [61]. In these systems, plant health is affected by the toxicity of contaminants and other environmental stresses, such as frequent fluctuation in water depth, which reduce biomass production and thus limits the purification process. Some endophytes have shown to reduce stress [2] and those with PGP activity, such as the water mint isolates SA, SB and SS, may improve the process of phytodepuration through enhancing plant growth. Moreover, larger root areas, other than increase plant nutrient and contaminant absorption, may give greater chances of establishing beneficial associations with rhizobacteria [62]. Root growth modulation by fungal endophytes towards more efficient and stress tolerant plants would be therefore tested in watermint and other macrophytes to withstand the pollutant loading and the stresses associated with these aquatic treatment systems. Future studies will be also addressed to the exploitation in field of fungal-related plant phenotypes to produce environmentally friendly bio-inoculants and enhance phytodepurative properties of plants.

## Supporting Information

S1 Dataset Effects of water mint endophytes: weights and morphometric data. (XLS)

S1 Fig The method used to measure root area and root depth of *Arabidopsis* plants grown *in vitro*. (TIF)

S1 Text Levels of IAA derivatives in cultures of *Cadophora luteo-olivacea* (SA). (DOCX)
