# Supplementary figures and images for: In Vitro Morphogenesis of Arabidopsis to Search for Novel Endophytic Fungi Modulating Plant Growth
Source: PLoS One. 2015 Dec 7;10(12):e0143353. doi: 10.1371/journal.pone.0143353 (PMC4671684; doi:10.1371/journal.pone.0143353)

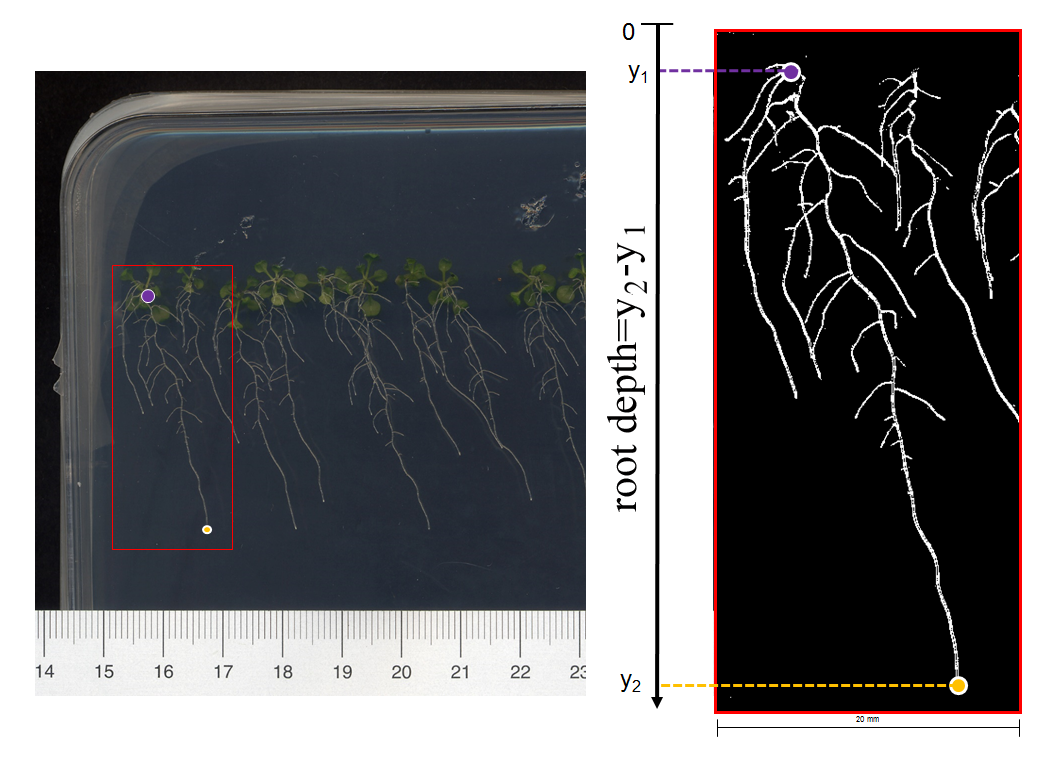

Supplement: S1 Fig — (TIF) [file pone.0143353.s002.tif]
