# Supplementary material for: In Vitro Morphogenesis of Arabidopsis to Search for Novel Endophytic Fungi Modulating Plant Growth
Source: PLoS One. 2015 Dec 7;10(12):e0143353. doi: 10.1371/journal.pone.0143353 (PMC4671684; doi:10.1371/journal.pone.0143353)
Supplement: S1 Text — (DOCX) [file pone.0143353.s003.docx]

**S1 Text. Levels of IAA derivatives in cultures of *Cadophora luteo-olivacea* (SA).**

**Materials and methods**

Two mycelial plugs were used to inoculate three flasks for each fungal endophyte containing 100 ml of M102 growth medium [1] without the addition of antibiotics. For each treatment set, one flask containing the growth media was used as a negative control. Flasks were incubated under agitation at 160 rpm at 25 ±1°C and checked for contamination after 72 h. After ten days of culture, three×1ml aliquots were removed from each flask and centrifuged at 2500 rpm per 5 min. A colorimetric technique was used to estimate the concentration of indole compounds by mixing the supernatant with 1 ml of Salkowski reagent (FeCl_3_ 0.138 M and H_2_SO_4_ 7.9 M) [2]. Samples were incubated at room temperature for 30 min in the dark, and then analyzed at 530 nm on a spectrophotometer (Beckman DU®530). IAA levels were determined with an IAA standard curve using commercial IAA (Sigma-Aldrich , St. Louis, MO) and sterile medium as a blank. IAA levels were expressed as µg/ml of growth medium and as µg/g of dry mycelium.

**Results and discussion**

None of the watermint endophytes produced IAA derivatives in liquid culture above the detection limit, with the exclusion of SA (*C. luteo-olivacea*), whose IAA-derivatives level was lower than the range of concentrations reported for other fungal endophytes. Production of IAA-derivatives also varied considerably among fungal isolates of the same species, i.e. compare IAA levels in SA and SL of *C. luteo-olivacea* (**Table**).

| **Table. IAA derivatives levels (µg/ml) in the growth medium of watermint fungal endophytes and in the fungal mycelia.** | | |
| --- | --- | --- |
| **Fungal acronyms** | **µg/ml** | **μg/g dry mycelium** |
| SA (*C. luteo-olivacea*) | 0.055 | 4.69 |
| SL (*C. luteo-olivacea*) | * | * |

* IAA values below method detection limits.

In literature, IAA levels reported in liquid cultures for other fungal endophytes range from 0.23 µg/ml to 71.51 µg/ml [3-5]. However, when the effect of different IAA levels were tested by [6], dose dependent effects on *in vitro* *Arabidopsis* growth (14 dai, MS medium) were found and significant increases of root and shoot fresh weights were obtained within the biologically active range of 0.011-0.003 µg/ml of pure IAA. This range encompasses the estimated level of SA. Besides, IAA level of SA (0.05 µg/ml) is higher than the average IAA production estimated by [6] for *T. virens* (0.014 µM) in absence of IAA precursors. However, as explained in “discussion” not all morphometric parameters were coherent with an effect of auxin, and alterations in RSA were similar to those induced by other watermint endophytes.

**References.**

1. Bacon CW, Porter JK, Robbins JD, Luttrell ES. Epichloe typhina from toxic tall fescue grasses. Appl Environ Microbiol. 1977; 34: 576-581.
2. Glickmann E, Dessaux Y. A critical examination of the specificity of the Salkowski reagent for indolic compounds produced by phytopathogenic bacteria. Appl Environ Microbiol 1995; 61: 793-6.
3. Fouda AH, Hassan SED, Eid AM, Ewais EED. Biotechnological applications of fungal endophytes associated with medicinal plant *Asclepias sinaica* (Bioss.). Ann Agric Sci. 2015; 60: 95–104.
4. Waqas M, Khan AL, Lee IJ. Bioactive chemical constituents produced by endophytes and effects on rice plant growth. J Plant Inter. 2014; 9: 478–487.
5. Waqas M, Khan AL, Kamra M, Hamayu M, Kang SM, Kim YH, et al. Endophytic fungi produce gibberellins and indoleacetic acid and promotes host-plant growth during stress. Molecules 2015; 9: 10754-10773.
6. Contreras-Cornejo HA, Macías-Rodríguez L, Cortés-Penagos C, López-Bucio, J. *Trichoderma vires*, a plant beneficial fungus, enhances biomass production and promotes lateral root growth through an auxin-dependent mechanism in *Arabidopsis*. Plant Physiol. 2009; 149: 1579–1592.
